# Adaptive knowledge distillation based structure-text embedding integrating for knowledge graph completion

**DOI:** 10.1371/journal.pone.0344363

**Published:** 2026-03-19

**Authors:** Qingsong Li, You Lv, Xiaolong Wei, Chao Li, Lianqiu Wei, Jianguang Zhang, Wei Wei

**Affiliations:** 1 School of Artificial Intelligence, Beihang University, Beijing, China; 2 Zhongguancun Laboratory, Beijing, China; 3 Key Laboratory of Mathematics Informatics Behavioral Semantics, Ministry of Education, Beijing, China; 4 Advanced Innovation Center for Future Blockchain and Privacy Computing, Beihang University, Beijing, China; 5 School of Mathematical Science, Beihang University, Beijing, China; 6 Department of Mathematics and Computer Science, Hengshui University, Hengshui, China; Philadelphia University, JORDAN

## Abstract

Knowledge graph completion (KGC) is a fundamental task for improving downstream applications like semantic search and question answering. Effective KGC requires integrating structural and description information, allowing them to complement each other’s weaknesses (e.g., long-tail issues or overlooked structural knowledge). Existing work typically integrates structural and description information at the embedding level by feeding structure embeddings into pre-trained language models (PLMs) and coupling them via attention mechanisms, which ensures the complementarity. However, as many KG entities are multi-semantic, exhibiting semantics beyond descriptions in certain triplets and making PLMs struggle to learn them, and current embedding level coupling approaches fail to transfer the entity multi-semantic knowledge learned from the structure model to PLM, the integration effect can be further improved. To alleviate above issue, we propose **AKD-KGC**, which aims at realizing this knowledge transfer then enhancing the integration effect by adding a teaching-learning procedure based on **A**daptive **K**nowledge **D**istillation during feature integration for **KGC** task in this work. The AKD-KGC framework integrates two features at the embedding level and use structural models to guide prediction behavior of integration model at the same time, adjusting the weight of PLM through additional supervision and enhancing its learning of entity additional semantics beyond descriptions. AKD-KGC can be applied to both transductive and inductive settings, and has achieved state-of-the-art results on a large number of datasets in both settings, demonstrating the effectiveness of our method. Our code and datasets are available at https://github.com/liqingsong1227/AKD-KGC.

## Introduction

Knowledge graph (KG) is a type of structured semantic knowledge base that is used to symbolically represent concepts within the physical world and their interconnectedness, which has extensive applications in various artificial intelligence applications, such as semantic search [1], question answering [2], and recommendation system [3,4]. Despite the inclusion of millions of entities and triplets, many KGs remain incomplete due to the continuous emergence of new knowledge. In order to address these challenges, researchers have focused their efforts on knowledge graph completion (KGC). Specifically, the KGC tasks can be categorized into transductive and inductive settings, depending on whether new entities appear in the test data.

KGC tasks can be formulated as predicting target value *v* when a query *q* is given, where the predicted value is selected through the plausibility of a (q,v) pair. Existing methods for computing plausibility can be roughly divided into two categories: structure-based and description-based, where structure-based methods concentrate more on learning embeddings that capture the global structure features of massive positive and negative query-value pairs with different score functions [5–7], while description-based methods utilize pre-trained language models (PLMs) to learn the contextual semantics from entity descriptions [8,9]. Structural methods always suffer from the long-tail issue, and description-based methods are limited by overlooking global structural information. Recently, many researchers explore the integration of both approaches to enable both types of information to autonomously play different role in different query scenarios and compensate for each other’s shortcomings [10–12]. They focus on achieving the integration of two types of information at the embedding level, by feeding structure embedding into PLM and dynamically coupling the two types of information through attention mechanisms, which achieves complementarity between two types of information.

**Fig 1:**
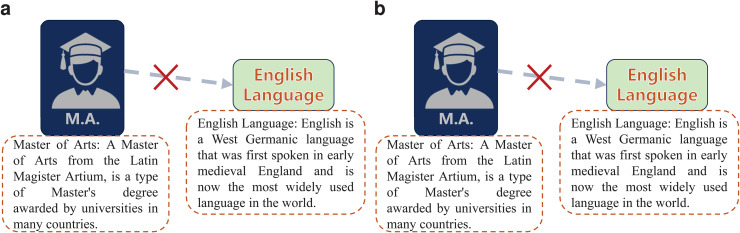
One example of multi-semantic entity. (**a**) The property of *English Language* from description does not match the given query. (**b**) The major field property from structure matches the given query. When answer the query (*Master of Arts, /education /educational degree /people with this degree. /education /education /major field of study,?*), entity *English Language* can not be matched through the description because the description only contains the semantic of language like sub-figure **(**a). However, in sub-figure **b**, entity *English Language* are connected with entity *Master of Science* with the same relation like other major fields such as *Linguistics*, which implies *English Language* can contain the semantics of major field.

However, these integrating methods have some limitations caused by the inherent shortcomings of PLMs when dealing with multi-semantic entities. Taking Fig 1 as an example, the correct value entities for query (*Master of Arts, /education /educational degree /people with this degree. /education /education /major field of study,?*) should be some major fields of study that are correspond with the master of arts degree. When using PLM to embed query and values, only the value entities whose description have some certain text about major field can have probability to satisfy this query. Unfortunately, there are many entities satisfy this query even though their descriptions have no certain text about major field, such as *English Language*, whose description only contain the information as a language, but they can still contain the semantic of a major field of study in some similar triplets like (*Master of Science, /education /educational degree /people with this degree. /education /education /major field of study, English Language*). In other words, there are many entities that exhibit semantics beyond their descriptions in certain triplets and these semantics are difficult for PLMs to learn. Although structure-based methods can acquire additional semantics via entity correlations, existing embedding-level approaches fail to fully leverage this knowledge. They restrict the use of multi-semantic knowledge to a supplementary role, preventing the transfer of knowledge to improve the PLM’s intrinsic prediction behavior. Addressing this limitation suggests that the performance of current methods can be substantially enhanced.

The major purpose of this work is realizing this multi-semantic knowledge transfer and enhancing the integration effect. At first, we compress the knowledge learned by the structural model into its scores for (q,v) pairs, where different scores represent the semantic relevance of different (q,v) pairs. For an entity, its matching scores with different queries contain its multi-semantic semantics. Next, we let the integration model learn the prediction behavior of structure model, i.e., learning to predict score of (q,v) pairs like structure model, so that although the description model cannot directly calculate the correlation outside the description semantics based on the description content, it can adjust the weight of PLM through the score of the structural model, making the PLM module of the fusion model can also learn this multi-semantic knowledge. Specifically, a novel structure and description integration framework named **AKD-KGC** are build, which not only integrate two features at the embedding level, but also add a teaching-learning procedure based on **A**daptive **K**nowledge **D**istillation during feature integration, to achieve multi-semantic knowledge transfer and guide the guide prediction behavior of integration model for **KGC** tasks. AKD-KGC employs a path-based GNN to capture KG structural features, then encodes queries and values separately using dual BERTs for descriptions, and finally fuses both embeddings. AKD-KGC employs a two-stage training process: first pre-training a structure-only GNN as the teacher model, then using adaptive knowledge distillation to train the integration model, enabling it to autonomously decide whether to learn from the teacher or data. AKD-KGC can be applied to both transductive and inductive settings, and has achieved state-of-the-art results on three transductive benchmark datasets, including WN18RR, FB15k-237, and CoDEx-M, and two inductive benchmark datasets with four standard splits including FB15k-237-V1 V4 and WN18RR-V1 V4, demonstrating the effectiveness of our method. The contribution of this work can be summarized as follows:

- A novel structure and description integration framework named AKD-KGC are build, which not only integrate two features at the embedding level, but also achieve multi-semantic knowledge transfer and guide the guide prediction behavior of integration model for KGC tasks.
- A teaching-learning procedure based on adaptive knowledge distillation is added during feature integration to achieve above knowledge transfer, enabling the integration model to autonomously decide whether to learn from the teacher or data.
- This AKD-KGC framework can be applied in both transductive and inductive setting and state-of-the-art on three transductive KGC datasets and two inductive KGC datasets with four standard splits are got.

## Related work

### Knowledge graph completion

Classic knowledge graph completion techniques mostly fall into two categories: structure-based techniques and recently developed description-based techniques.

### Structure-based methods

Structure-based methods use structure statistical characteristics to directly predict, or generate embedding for entities by directly defining an embedding matrix or using graph neural networks to aggregate message from their neighbors, then use appropriate scoring functions to compute probability for a triplet.

**Structure statistical characteristics** mainly include path features such as Path Ranking [13,14] which directly uses relational paths as symbolic features for prediction, NeuralLP [15] and DRUM [16] which learn probabilistic logical rules to weight different paths.

**Structure Embedding methods** learn the near neighbor semantic relationship between entities and are limited by the long-tail problem. These methods can be divided into three categories based on score functions: translation distance methods, semantic matching methods and neural network methods. The fundamental concept of the translation distance model involves conceptualizing the relation between a subject entity and an object entity as a form of translation, whereby the plausibility is determined by the distance of translation between the two entities. Some representative translation based methods include TransE [5], TransH [17], TransR [18], TransD [19] and Rotate [20]. Semantic matching model measures the plausibility of a triplet by matching latent semantics in the embedding space. Typical methods include RESCAL [6], Distmult [21], ComplEx [22], QutatE [23], DURA [24] and BLMSearch [25]. The neural network model builds a neural network to compute the plausibility of one triplet, and it includes ERMLP [26], NTN [27] and ConvE [28], DiffusionE [29]. To better learn the structure feature of KG, some researchers use graph neural networks to generate more compressive embeddings, such as R-GCN [30], HRAN [31], DisenKGAT [32], Path-RNN [33], NBFNet [7], StructKGC [34].

### Description-based methods

The first description-based KGC methods DKRL [35] uses a convolution neural network to embed entity’s descriptions. With the development of natural language processing, large pre-trained language models such as GPT1/2/3 [36–38], BERT [39], T5 [40] become mainstream encoder of natural sentences. Description based methods also benefit from these language models, such as KG-BERT [8], LMKE [9], SimKGC [41], MoCoKGC [42] and Csprom-KD-DC [43], use language models as entity and description encoder and get prior knowledge from pre-trained weights. Due to the large cost of fine-tuning BERT, these methods are limited by the number of negative samples.

Recently, LASS [10], CSprom-KG [11] and Mocosa [12] explore the fusion of structure and description approaches and adopt paradigms where the output of structural models (e.g., TransE [5]) is fed into PLMs like BERT as part of the input. However, these paradigms can not be used in inductive setting and have limitations on diluting structure information.

### Knowledge distillation

The main idea of knowledge distillation is that the student model mimics the teacher model in order to obtain a competitive or even a superior performance, and it has received increasing attention from the research community in recent years. It has achieved remarkable success with good performance in many fields such as model compression and acceleration [44], multi-modal learning [45] and knowledge graph completion [46]. More introduction about knowledge distillation can be seen in the survey [47].

## Materials and methods

In this section, the basic concepts, notation and the details of our proposed framework will be introduced.

Concepts and Notations

- **Knowledge Graph and Knowledge Graph Embedding.** A knowledge graph(KG) can be described as a tuple 𝒢=(𝒱,ℛ,ℰ), where 𝒱 denotes the entity set, ℛ denotes the relation set, and ℰ⊆𝒱×ℛ×𝒱 denotes the triple set. A triple (u,r,v)∈ℰ denotes a fact or knowledge in KG, where *u*, *v* denotes head and tail entity respectively, and *r* denotes relation between them. Knowledge graph embedding aims at learning or calculating a vector representation for each entity and relation, then uses this vector representation for downstream tasks such as knowledge graph completion and triplet classification. In this paper, we use bold terms to denote vector representations, such as u to denote vector representation of entity *u*, and E to denote vector representations of all entities ℰ.
- **Knowledge Graph Completion.** Knowledge graph completion aims at answering two queries (u,r,?) and (?,r,v), where the second one is changed into the query $\left(v,r^{-1},?\right)$, in which $r^{-1}$ denotes the reverse relation of relation *r*. We use q=(u,r) to represent a specific query (u,r,?). Researchers use a score function f(u,r,v) to calculate plausibility for all candidate values of a given query and make predictions based on them.

### AKD-KGC overview

**Fig 2:**
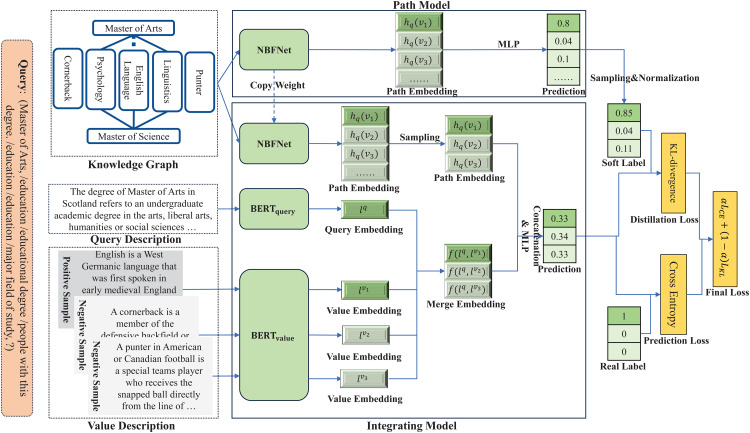
The architecture of our AKD-KGC framework. We aim to train a model to answer the query (*Master of Arts, /education /educational degree /people with this degree. /education /education /major field of study,?*). The left is the input features, including paths in KG, descriptions of query and value, where for a positive value, we sample several negative samples for learning of integrating model. The upper half in the middle is a pre-trained NBFNet, while the lower half in the middle is our integrating model, including an NBFNet that is loaded from the pre-trained one, and two BERT to encode query and values with descriptions. The right shows the loss function of our integrating model, including prediction loss and distillation loss, where two losses are dynamically weighted sum.

Specifically, we use NBFNet [7] to learn structural representations containing different paths between query and value, and learn description embeddings of query and value separately by using two BERTs. Then we integrate two embeddings and make predictions based on the integrated embedding. To achieve multi-semantic knowledge transfer and guide the guide prediction behavior of integration model, we adopted a two-stage training process. Firstly, we trained an NBFNet to provide an initial output logits as soft label. Subsequently, when training the integrating model, we not only let the integrating model learn directly from the data, but also let the integrating model learn from the pre-trained NBFNet through knowledge distillation. Additionally, in order to prevent the integrating model from being limited by the performance of the NBFNet, we used an adaptive mechanism to adaptively balance learning from the teacher and the ground truth, ensuring it avoids suboptimal convergence due to noisy input while maintaining its own performance capacity. The overall framework is shown in Fig 2.

## Components of integrating model

### Description module

In order to utilize the description semantics of a triplet, we use two pre-trained BERT models to learn the query and value embeddings separately. The description of an entity *u* (or a relation *r*), which can be quired from original database, is a sequence of tokens $d_{u}=(x_{n_{1}},...,x_{n_{u}})$ that gives a detailed introduction for the entity or relation, where $n_{u}$ denotes the length of the tokens, $\{x_{i}^{u}{\}}_{i=1}^{n}$ denotes the description tokens of entity *u*. For the embedding of query q=(u,r), the input tokens $t_{q}$ of pre-trained BERT model are joined from entity token and description of entity *u* and relation *r* following with a special token [CLS], that is $t_{q}=(x_{u},d_{u},x_{r},d_{r},\text{[CLS]})=(x_{u},x_{1}^{u},...,x_{n_{u}}^{u},x_{r},x_{1}^{r},...,x_{n_{r}}^{r},\text{[CLS]})$, where $x_{u}$ denote the token of entity *u* itself. Benefiting from the powerful contextual understanding ability of BERT model, every query can gain a vector representation containing rich description semantics. Although some entities or relations may lack description, they can still learn representation from related entity’s or relation’s description. We use the output embedding of token [CLS] as the final embedding of query *q* and recorded as $l^{q}$, which aggregates all information of whole tokens through attention mechanism. Embedding for value *v* can be calculated similarly. The input token of value *v* is $t_{v}=(x_{v},d_{v},\text{[CLS]})=(x_{v},x_{1}^{v},...,x_{n_{v}}^{v},\text{[CLS]})$, then we use the embedding of token [CLS]  as the final embedding of value *v*, and it is recorded as $l^{v}$.

### Path module

We solve the path representations by NBFNet [7], which uses the generalized Bellman-Ford Algorithm to overcome the exponential quantity issue. NBFNet defines the path semantics between query q=(u,r) and value *v* as a *generalized* *sum* of path representations between *u* and *v* with a commutative *summation* operator ⊕, where each path representation can be defined as a *generalized* *product* of the edge representations in the path with the *multiplication* operator ⊗. It can be formulated as follows:


$$\begin{array}{c} h_{q}(v)=h_{q}(P_{1})⊕h_{q}(P_{2})⊕\cdots ⊕h_{q}(|{𝒫}_{u,v}|)=\underset{P\in {𝒫}_{uv}}{⊕}h_{q}(P), \end{array}$$ (1)

$$\begin{array}{c} h_{q}(P=(e_{1},...,e_{\left|P\right|}))=w_{r}(e_{1})⊗w_{r}(e_{2})⊗\cdots ⊗w_{r}(e_{\left|P\right|})=\overset{\left|P\right|}{\underset{i=1}{⊗}}w_{r}(e_{i}), \end{array}$$ (2)

where ${𝒫}_{uv}$ represents all paths between *u* and *v* and $w_{r}(e_{i})$ represents the edge representation of edge $e_{i}$. This path formulation can be solved by Generalized Bellman-Ford Algorithm and parameterized three operators. Specifically, given a query q=(u,r), the conditional representation of each target node *v* with *q* can be initialized through an INDICATOR operator, then the conditional representation of each node can be updated with Bellman-Ford iteration, like


$$h_{v}^{\left(0\right)}=\text{INDICATOR}(v,q)\;h_{v}^{\left(l\right)}=\text{AGGREGATE}(\{\text{MESSAGE}(h_{x}^{\left(l-1\right)},w_{r}(x,r,v))|\;(x,r,v)\in E(v)\}\cup \{h_{v}^{\left(0\right)}\}),$$ (3)

where l∈{1,2,⋯,L} and *L* is the total layer of NBFNet. The INDICATOR operator is defined as $\text{INDICATOR}(v,q)={𝕀}_{u}(v)\times r$, in which ${𝕀}_{u}(v)=1$ if v=u otherwise 0. The MESSAGE operator is translation or scaling operators such as the relational operators used in TransE [5] and DistMult [21], and $w_{r}(x,r,v)=W_{r}r+b_{r}$ represents the conditional edge representation. The AGGREGATE operator can be instantiated as natural summation, max or min in traditional methods, and we used the principal neighborhood aggregation (PNA) proposed in [48]. It should be noted that all the node representations $h_{v}^{\left(t\right)}$ in equation (3) are conditioned with query *q*, and they are pair representations of *u* and *v*, rather than single node representations. The path semantics between *u* and *v* with query *q* are embedded in the final layer $h_{v}^{\left(L\right)}$, and we record it as $h_{q}(v)$.

### Integrating module

We first fuse two kinds of semantic features by directly concatenating semantic embeddings, and we introduce additional node degree information for final prediction. We feed the concatenated embedding into a two layer MLP and the probability that *q* matches *v* is


$$p_{s}(q,v)=\sigma (\text{MLP}[f(l^{q},l^{v});d;h_{q}(v)]),$$ (4)

where $d=[\log(d_{u}+1);\log(d_{v}+1)]$, σ(· represents sigmoid activation function, f(·) represents a merging function which can be instantiated as difference, multiplication, concatenation or their composition, and [·;·] represents concatenation operator.

### Training process

We use weighted Binary Cross Entropy(BCE) as loss of learning from data, like


$$\begin{array}{c} \begin{array}{cc} {ℒ}_{BCE}= & -\sum_{i}[w_{i}(y_{i}\log p_{s}(q,v_{i})+(1-y_{i})\log(1-p_{s}(q,v_{i})))], \end{array} \end{array}$$ (5)

where


$$\begin{array}{c} w_{i}=\text{softmax}(p_{s}(q,v_{i}))=\frac{e^{p_{s}(q,v_{i})}}{\sum_{j}e^{p_{s}(q,v_{j})}}, \end{array}$$ (6)

and *i* is selected from all positive and negative value samples for a given query.

In the training stage, we first pre-train an NBFNet, then regard our integrating model as student model and use the pre-trained NBFNet as teacher model. We use adaptive knowledge distillation to help training student model with the soft labels from the teacher, where the weight of path module in student model is directly loaded from the pre-trained NBFNet for less back propagation.

Let the normalized output logits of student model and teacher model is $\phi_{s}$ and $\phi_{t}$, then the distillation loss can be described as the KL-divergence between $\phi_{s}$ and $\phi_{t}$. We use the average of $KL(\phi_{s};\phi_{t}$ and $KL(\phi_{t};\phi_{s})$ as our final distillation loss, like


$${ℒ}_{KL}=\frac{1}{2}(KL(\phi_{s};\phi_{t})+KL(\phi_{t};\phi_{s})),$$ (7)

where the KL-divergence is defined as


$$KL(p_{1};p_{2})=\int p_{1}(x)\log\frac{p_{1}(x)}{p_{2}(x)}dx,$$ (8)

and $p_{1}$, $p_{2}$ are two arbitrary distribution.

Finally, the integrating model has much better potential in getting good performance due to it incorporates more description information than teacher model, so distilling directly from the teacher model with a fixed coefficient is suboptimal. Accordingly, we use the performance of student and teacher models to adaptively adjust weights of student models learning from ground truth or soft labels. Specifically, we use the Cross Entropy(CE) between prediction results and ground truth of student model or teacher model as performance indicator, then we use softmax function to normalize them. The CE can be written as


$$\begin{array}{c} CE_{s}=-\sum_{i}[y_{i}\log p_{s}(q,v_{i})+(1-y_{i})\log(1-p_{s}(q,v_{i}))], \end{array}$$ (9)

$$\begin{array}{c} CE_{t}=-\sum_{i}[y_{i}\log p_{t}(q,v_{i})+(1-y_{i})\log(1-p_{t}(q,v_{i}))]. \end{array}$$ (10)

The final loss is


$$ℒ=\alpha \cdot L_{BCE}+(1-\alpha )\cdot L_{KL},$$ (11)

where


$$\alpha =\frac{e^{-CE_{s}/\tau }}{e^{-CE_{s}/\tau }+e^{-CE_{t}/\tau }},$$ (12)

and *τ* is a hyperparameter controlling the influence of CE value to selecting weight.

## Experimental setting

### Datasets

We evaluated our method in both transductive and inductive knowledge graph completion. For transductive KGC, we used three competitive benchmark datasets: FB15k-237 [49], WN18RR [28], and CoDEx Medium [50], and we used standard transductive splits [28,49,51]. For inductive KGC, we followed splits in [52] for FB15k-237 and WN18RR. Statistics of these datasets are shown in Table 1 and Table 2.

**Table 1:** Statistics of Datasets in Transductive Setting.

Datasets		WN18RR	FB15k-237	CoDEx-M
# Entities		40,493	14,541	10,750
# Relations		11	237	51
# Edges	# Train	86,835	272,115	185,584
	# Valid	3,034	17,535	10,310
	# Test	3,134	20,466	10,311
# Mean Degree		2.12	18.71	12.09

**Table 2:** Statistics of datasets in inductive setting. Queries refer to the triplets that are used as training or test labels, while facts are the triplets used as training or test inputs. In the training sets, all queries are also provided as facts.

Dataset		# Relation	Train				Valid			Test			
			# Entity	# Query	# Fact	# Mean-d	# Entity	# Query	# Fact	# Entity	# Query	# Fact	# Mean-d
FB15k-237	v1	180	1,594	4,245	4,245	2.66	1,594	489	4,245	1,093	205	1,993	1.82
	v2	200	2,608	9,739	9,739	3.73	2,608	1,166	9,739	1,660	478	4,145	2.50
	v3	215	3,668	17,986	17,986	4.90	3,668	2,194	17,986	2,501	865	7,406	2.96
	v4	219	4,707	27,203	27,203	5.78	4,707	3,352	27,203	3,051	1,424	11,714	3.85
WN18RR	v1	9	2,746	5,410	5,410	2.24	2,746	630	5,410	922	188	1,618	1.5
	v2	10	6,954	15,262	15,262	2.19	6,954	1,838	15,262	2,757	441	4,011	1.45
	v3	11	12,078	25,901	25,901	2.14	12,078	3,097	25,901	5,084	605	6,327	1.24
	v4	9	3,861	7,940	7,940	2.06	3,861	934	7,940	7,084	1,429	12,334	1.74

### Baselines

We compare our methods against path-based methods, structure-based methods, GNN-based methods, description-based methods and combination-based methods for transductive setting, which includes 16 baselines. For inductive setting, we compare against path-based methods and GNN-based methods, which includes 5 baselines.

### Implementation details

Our implementation generally follows the open source codebases of knowledge graph completion. We augmented each triplet (u,r,v) with a flipped triplet $\left(v,r^{-1},u\right)$. We used the original setting in [7] for pre-training the teacher NBFNet, and used BERT-base as our description module. The learning rate of fine-tuning PLM and other components were 2e−5 and 1e−3 respectively. Batch size was set as 24 for WN18RR, 48 for FB15k-237 and CoDEx-M. Our student model was trained on 1 Tesla A100 GPUs for 12 epochs, and we selected the models based on their performance on the validation set.

### Evaluation metrics

For transductive setting, we used the same setting as much previous work such as [30–32]. Specifically, given the triplet in the test set, we ranked it by the value of score function *f* against all candidate triplets which filter out all the correct triplets appearing in the training, validation, and test datasets, and it’s the same as other work for fair comparison. We used three kinds of metrics including mean rank(MR), mean reciprocal rank(MRR), and Hits@*k*(for k=1,10). For inductive relation prediction, we followed [52] to draw 50 negative triplets for each positive triplet and use the above filtered ranking. We report HITS@10 for comparison.

## Experimental results

### Main results

Table 3 summarizes the results on transductive knowledge graph completion. AKD-KGC performs better than baseline methods on all three datasets and nearly all metrics. Compared with other integrating-based methods, AKD-KGC get significant improvements.

**Table 3:** Transductive knowledge graph completion results. The results of NBFNet and LMKE on Codex Medium are trained with original experimental setting and other results are directly taken from the original papers and some results are omitted because the relevant code has not been open sourced. The best results are in bold and the second-best results are underlined.

Class	Method	FB15k-237				WN18RR				Codex Medium			
		MR	MRR	H@1	H@10	MR	MRR	H@1	H@10	MR	MRR	H@1	H@10
Path Based	Path Ranking	3521	0.174	0.119	0.285	22438	0.324	0.276	0.406	–	–	–	–
	NeuralLP	–	0.240	–	0.362	–	0.435	0.371	0.566	–	–	–	–
	DRUM	–	0.343	0.255	0.516	–	0.486	0.425	0.586	–	–	–	–
Structure Based	TransE	357	0.294	–	0.465	3384	0.226	–	0.501	–	0.303	0.223	0.454
	ComplEx	339	0.247	0.158	0.428	5261	0.44	0.41	0.51	–	0.337	0.262	0.476
	ConvE	244	0.325	0.237	0.501	4187	0.43	0.40	0.52	–	0.318	0.239	0.464
	TuckER	–	0.358	0.266	0.544	–	0.470	0.443	0.526	–	0.328	0.259	0.458
GNN Based	R-GCN	–	–	–	–	–	0.249	0.151	0.428	–	–	–	–
	HRAN	156	0.355	0.263	0.541	2113	0.479	0.450	0.542	–	–	–	–
	DisenKGAT	179	0.368	0.275	0.553	1504	0.486	0.441	0.578	–	–	–	–
	NBFNet	114	0.415	0.321	0.599	636	0.551	0.497	0.666	312	0.367	0.284	0.525
Description Based	LMKE	141	0.306	0.218	0.484	78	0.619	0.523	0.789	453.4	0.261	0.196	0.388
	SimKGC	–	0.336	0.249	0.511	–	0.666	0.587	0.800	–	–	–	–
Integration Based	LASS	**108**	–	–	0.533	**35**	–	–	0.786	–	–	–	–
	CSProm-KG	–	0.358	0.269	0.538	–	0.575	0.522	0.678	–	–	–	–
	SEA-KGC	–	0.367	0.275	0.553	–	0.653	0.577	0.795	–	–	–	
	**AKD-KGC** (Ours)	114	**0.419**	**0.327**	**0.602**	112	**0.692**	**0.601**	**0.854**	**307**	**0.369**	**0.285**	**0.527**

In general, integrating-based methods and GNN-based methods outperform other methods on FB15k-237 and Codex Medium datasets, while integrating-based methods and description-based methods are better than other methods on WN18RR dataset. We assume that the FB15k-237 and Codex Medium have high average degrees, which means structure model can learn more structure features, while the WN18RR has lower average degree and its entities and relations are almost common noun and their facts are almost language knowledge, which is easy for language models to learn. By integrating description semantics, AKD-KGC gets additional improvements compared with previous SOTA method NBFNet [7] on FB15k-237 and Codex Medium, and we think that they lead a small margin is due to the difficulties on fine-tuning BERT model in these two datasets. The description module of AKD-KGC is similar to LMKE [9] and AKD-KGC improves 11% of MRR on WN18RR compared to LMKE and this proves the validity our structure features and integrating module.

Unlike other integration-based methods, AKD-KGC can be used in inductive setting. Table 4 shows all results on inductive setting. On WN18RR datasets, our method outperforms all other methods on all splits, and on FB15k-237, our methods outperform all other methods on three splits. The experimental results show that adding description features could improve more performance for graphs with lower average degree.

**Table 4:** Inductive knowledge graph completion results(HITS@10). The v1-v4 correspond to the 4 standard versions of inductive splits. Results of compared methods are taken from [7]. The best results are in bold and the second best results are underlined.

Class	Method	FB15k-237				WN18RR			
		v1	v2	v3	v4	v1	v2	v3	v4
Path Based	NeuralLP	0.529	0.589	0.529	0.559	0.744	0.689	0.462	0.671
	DRUM	0.529	0.587	0.529	0.559	0.744	0.689	0.462	0.671
	RuleN	0.498	0.778	0.877	0.856	0.809	0.782	0.534	0.716
GNN Based	GraIL	0.642	0.818	0.828	0.893	0.825	0.787	0.584	0.734
	NBFNet	0.834	0.949	**0.951**	0.960	0.948	0.905	0.893	0.890
Integration Based	**AKD-KGC** (Ours)	**0.917**	**0.951**	0.947	**0.962**	**0.982**	**0.977**	**0.955**	**0.964**

### Analysis

We conduct ablation study to show the effectiveness of our proposed components on three transductive KGC datasets. In order to prove the necessity of each semantic feature, we trained a model without description module (AKD-KGC w/o description) and a model without path semantic module (AKD-KGC w/o path). We also trained a model without distillation loss (AKD-KGC w/o distillation) to prove the necessity of adaptive knowledge distillation and a model without path module but with distillation loss(AKD-KGC-description w distillation) to prove the necessity of feature integration. We report the MRR and Hits@1 metrics for comparison and all models are trained with same setting.

Table 5 summarizes all ablation study results, and we set the complete model as baseline. In WN18RR datasets, dropping any module will make the performance decrease significantly, and the performance of AKD-KGC-description w distillation is much worse than the baseline. This indicates that both description module and path module could learn one aspect of knowledge graph semantics and the importance of distillation. Additionally, the performance decrease of dropping distillation module compared to baseline and the performance increase of adding distillation compared to AKD-KGC w/o path prove that distilling from path module is benefit for language module learning, which further improves the importance of multi-semantic knowledge transfer. While in FB15k-237 and CoDEx Medium datasets, dropping description module only makes the performance decrease slightly, and dropping path module makes the performance decrease dramatically. This indicates that our description module does not fully learn the natural semantics of entities and relations and we think this is due to fine-tuning BERT for such events and proper nouns is still challenging and it worths for further studying, and the performance of AKD-KGC w/o distillation is even worse than AKD-KGC w/o description further illustrates this issue.

**Table 5:** Ablation study results of transductive KGC.

Method	WN18RR			FB15k-237			CoDEx Medium		
	MRR	H@1	H@10	MRR	H@1	H@10	MRR	H@1	H@10
AKD-KGC	0.692	0.601	0.854	0.419	0.327	0.602	0.369	0.285	0.527
AKD-KGC w/o description	0.549	0.497	0.657	0.417	0.325	0.600	0.367	0.284	0.525
AKD-KGC w/o path	0.428	0.270	0.745	0.287	0.207	0.459	0.261	0.196	0.386
AKD-KGC-description w distillation	0.506	0.359	0.772	0.302	0.221	0.480	0.270	0.203	0.392
AKD-KGC w/o distillation	0.664	0.579	0.831	0.369	0.276	0.557	0.275	0.217	0.383

We then analyse the effect of weight of learning from teacher model or ground truth to the performance on WN18RR dataset. We conduct this by setting different values of hyperparameter *τ*. If *τ* is set large enough, student model will learn from teacher model with a fixed coefficient, and if *τ* is set small, student model will learn from teacher model with a dynamic coefficient. Specifically, when the performance of the student model is better than that of the teacher model, the student model tends to learn directly from the data and reduce the weight learned from the teacher model. We set five different values of *τ*: 1e+10, 1, 0.1, 0.01 and 1e−10. The experimental results are summarized in Table 6. Results show that our adaptive learning strategy does improve the performance and with smaller *τ*, student model could perform better.

**Table 6:** Parameter analysis of *τ* on WN18RR.

*τ*	MRR	H@1	h@10
1e+10	0.685	0.594	0.840
1	0.690	0.599	0.850
0.1	0.692	0.601	0.854
0.01	0.691	0.603	0.852
1e-10	0.691	0.602	0.853

### Detailed results

**Fig 3:**
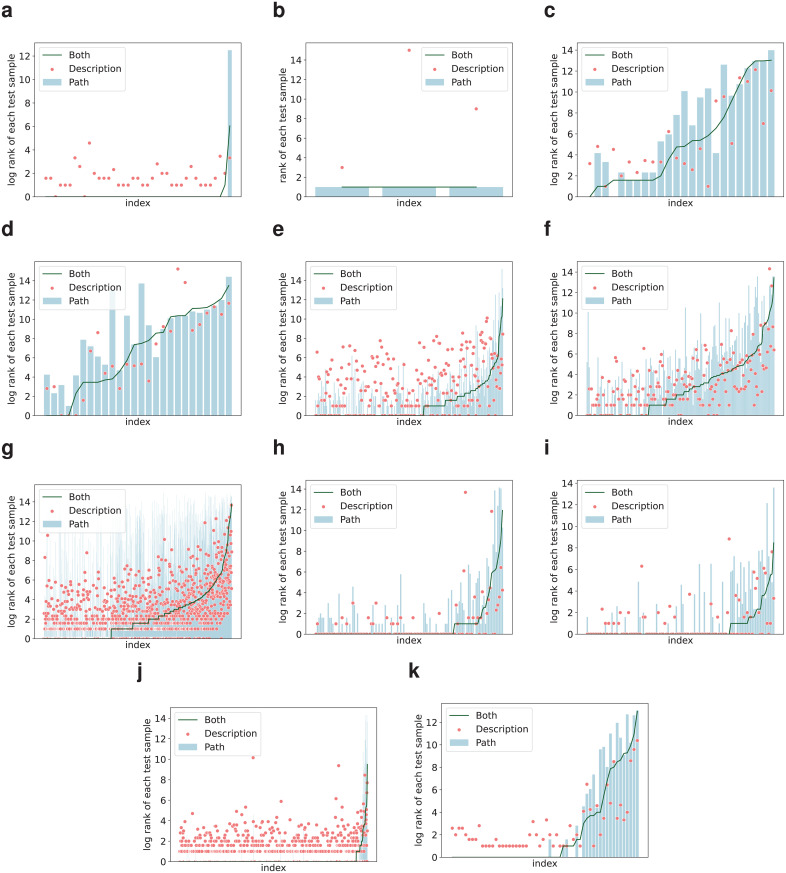
Rank values of each sample in test set, where these values are grouped by relation type. (**a**) *_verb_group*. (**b**)*_similar_to*. (**c**) *_member_of_domain_usage*. (**d**) *_member_of_domain_region*. (**e**) *_member_meronym*. (**f**) *_has_part*. (**g**) *_hypernym*. (**h**) *_instance_hypernym*. (**i**) *_synset_domain_topic_of*. (**j**) *_derivationally_related_form*. (**k**) *_also_see*.

In this section, to understand how does our AKD-KGC framework perform better than a single feature model, the detailed prediction results of three different ablation models are visualized, where three models include AKD-KGC (**Both**), AKD-KGC w/o description(**Path**) and AKD-KGC-description w distillation (**Description**). The complex relations in KG can be classified into 1-to-1, 1-to-N, N-to-1, and N-to-N [31] and the MR of each category on WN18RR of are summarized in Tabel Additionally, the rank of each test sample in each relation is shown in Fig 3.

The green line represents the overall integrating model values, the red scatter point represents the description module values and the light blue bar represents path module values.

The Fig 3 results show that the whole integrating model performs better than single path-based model in all relations, which improves that integrating description feature could supplement the losses caused by using only structural features, except some relation like *_member_of_domain_region*, *_hypernym* and *_also_see*, where the whole integrating model performs worse than single description-based model. The graph further demonstrating this phenomenon. We think this is caused by the lack of path features and the distillation process mislead the learning of the description module to some extent.

## Discussion

The experimental results demonstrate the effectiveness of our proposed AKD-KGC framework. By integrating structure and description features, and using adaptive knowledge distillation, our framework achieves state-of-the-art performance on both transductive and inductive KGC tasks. The ablation studies confirm the importance of each component in our framework, highlighting the benefits of multi-semantic knowledge transfer. Compared with existing methods, AKD-KGC shows significant improvements, especially on datasets with lower average degrees, where description features play a crucial role. Overall, our framework provides a promising direction for future research in knowledge graph completion by effectively combining multiple semantic sources.

### Limitations

This Work has some limitations. First, the fine-tuning of BERT for entities and relations that are proper nouns or events remains challenging, which may limit the effectiveness of the description module in certain datasets. Second, integrating knowledge graph structure with large language models is an area that requires further research, especially in light of recent advancements in foundation models across various domains. Finally, since foundation models have been widely investigated in other fields such as computer vision and natural language processing, building foundation model for KGC task with considering integrating two semantics is also worthy for future studying. In future work, we aim to establish a stronger theoretical foundation for the adaptive distillation mechanism. Specifically, we plan to analyze the correlation between the adaptive weights and information transformation from an information-theoretic perspective, providing mathematical guarantees for the model’s convergence and generalization ability.

### Threats to validity

This study may have several threats to validity. The choice of datasets, while standard in the field, may not fully represent the diversity of real-world knowledge graphs, potentially limiting the generalizability of our findings. Additionally, the hyperparameter settings and model architectures were selected based on prior work and may not be optimal for all scenarios. The reliance on pre-trained models like BERT also introduces dependencies on their training data and biases, which could affect the performance of our framework. Finally, while we conducted extensive experiments, there may still be unexamined factors that could influence the results, such as different training regimes or alternative integration strategies.

## Conclusion

In this paper, to achieve multi-semantic knowledge transfer from structure to description model and guide the guide prediction behavior of integration model, we built a novel structure and description integration framework named AKD-KGC, which not only integrate two features at the embedding level, but also add a teaching-learning procedure based on adaptive knowledge distillation during feature integration. We conducted adequate experiments on both transductive and inductive KGC benchmarks, and got state-of-the-art results in both settings, which improved the effectiveness of our framework.Furthermore, it is worth noting that the benefits of text-structure integration are correlated with the semantic richness of the textual information. As observed, the performance gains may vary across datasets, showing more significant improvements in scenarios with rich descriptions compared to datasets dominated by proper nouns where textual semantics are limited.
